# Autonomic hyperreflexia after spinal cord injury managed successfully with intravenous lidocaine: a case report

**DOI:** 10.1186/s13037-016-0098-5

**Published:** 2016-03-15

**Authors:** Pedro Leão, Paulo Figueiredo

**Affiliations:** Department of Anesthesiology and Pain Medicine, Centro Hospitalar de Entre o Douro e Vouga, Rua Dr. Cândido Pinho, 4520-211 Santa Maria da Feira, Portugal; Director of the Department of Anesthesiology and Pain Medicine, Centro Hospitalar de Entre o Douro e Vouga, Santa Maria da Feira, Portugal

**Keywords:** Autonomic hyperreflexia, Spinal cord injury, Patient safety, Intravenous lidocaine

## Abstract

**Background:**

Some paraplegic patients may wish undergo some surgical procedures, like urological procedures, without anesthesia. However, these patients can develop autonomic hyperreflexia if cystoscopy is performed without anesthesia.

**Case presentation:**

We present a case of severe autonomic hyperreflexia in a 44-year-old male with spinal cord injury at the level of T4 during urologic procedure under sedation and analgesia successfully treated with intravenous lidocaine.

**Conclusions:**

This case illustrates that patients with spinal cord injuries are likely to develop autonomic hyperreflexia during urological procedures performed without anesthesia. Health professionals should educate spinal cord injury patients regarding risks of this serious condition and be aware to prevent and manage autonomic hyperreflexia. In an acute episode, nifedipine, nitrates and captopril are the most commonly used and recommended agents. To our knowledge, this is the first case report of severe autonomic hyperreflexia treated successfully with intravenous lidocaine.

## Background

Autonomic hyperreflexia (AHR) is a potentially life-threatening hypertensive condition that develops in up to 85 % of patients with spinal cord injury (SCI) above the splanchnic outflow, usually above the level of T6 [1–4]. It is commonly triggered by afferent stimuli below the level of injury, such as distension of hollow viscera (bladder, uterus, gallbladder,bowel), uterine contractions during obstetric delivery, cutaneous stimulation, and surgical procedures involving pelvic organs or lower extremities [5, 6]. Clinical manifestations of AHR include marked hypertension, bradycardia, cardiac dysrhythmias, headache, piloerection, sweating, and flushing above the level of the lesion [7]. The severe hypertension may lead to seizures or fatal cerebral hemorrhage [8]. If not recognized as a medical emergency and promptly treated, acute AHR may result in devastating complications [9]. We presente a case of severe AHR in a patient with SCI at level of T4 during an urologic procedure under sedation and analgesia, treated sucessfully with intravenous lidocaine.

## Case presentation

**Fig. 1:**
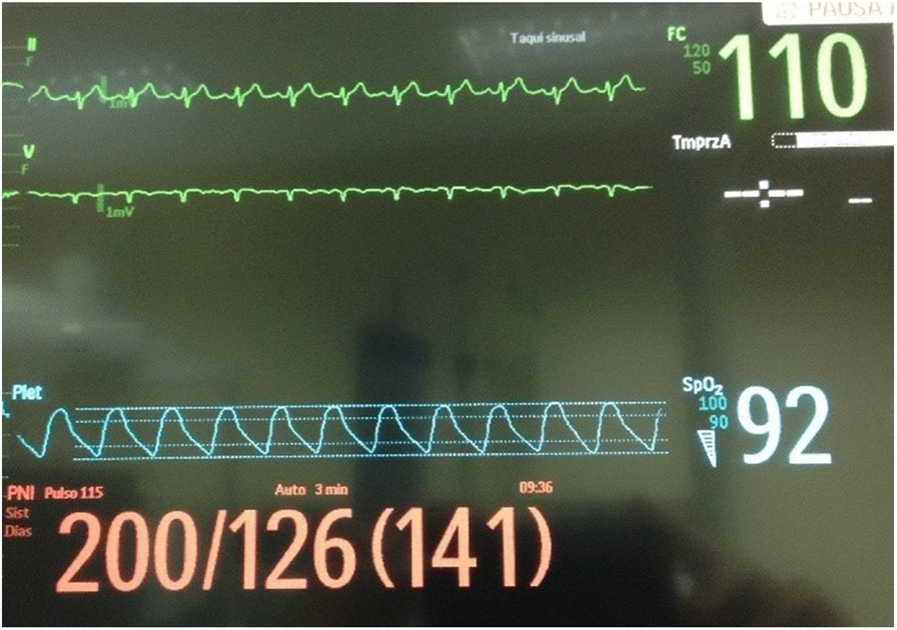
Severe hypertension after distension of the bladder. Clinical manifestations of AHR include marked hypertension. In this case, severe bradycardia (30 bpm) occurs and atropine was given. Blood pressure continues to rise and tachycardia occurs

**Fig. 2:**
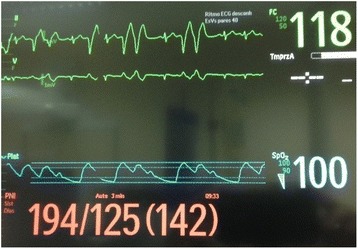
Ventricular bigeminy. Cardiac dysrhythmias is one of the clinical manifestations of AHR

A 44-year-old male, with a SCI at the level of T4, was scheduled for elective cystoscopy. This patient was managing his bladder by indwelling catheter. Urethral catheter got blocked frequently. The patient had no history of AHR in several years before this surgery and he undergone three operations before, the last one under sedation and analgesia, without reported problems. In the operating room, patient was reluctant to have general anesthesia, because he belief that anesthesia lead to complications and his stay in the hospital could be prolonged. The subarachnoid block was technically difficult in one previous surgery (same surgery) and he had thrombocytopenia (98 × 10^9^/L). Patient underwent sedation and analgesia with intravenous midazolam 2 mg, fentanyl 0,1 mg, paracetamol 1 g and ketorolac 30 mg. Prior to the introduction of the cystoscopy, blood pressure (BP) was 131/76 mmHg and heart rate (HR) was 78 bpm. After distension of the bladder, BP increased to 194/125 mmHg and severe bradycardia occurs (30 bpm). Atropine 0.5 + 0.5 mg (total 1 mg) was given. BP continues to rise to 200/126 mmHg (Fig. 1) and tachycardia occurs with ventricular bigeminy (Fig. 2). The patient complained of severe headache. Fentanyl 0,1 mg + midazolam 2 mg were administered. Therefore, a intravenous bolus of lidocaine (1.5 mg/kg) was given. About 3 min later, there was complete resolution of symptoms, with BP and HR return to baseline. The surgery lasted 25 min and the patient went to the post-anesthesia care unit (PACU) hemodynamically stable. There were no complications during the permanence in the PACU. He was discharged home in first postoperative day, and no sequelae were noted.

## Conclusions

This case reports a serious condition in a SCI patient with a lesion at the level of T4, demonstrating that these patients are likely to develop AHR during cystoscopy performed under sedation and analgesia. Anesthesiologists should discuss and educate SCI patients regarding risks of AHR and possible life-threatening complications, when urologic procedures like cystoscopy are carried out without anesthesia. If spinal patients are made aware of these serious complications of AHR, they are less likely to decline anesthesia for urological procedures.

It has been observed that, the higher the injury level, the greater the degree of clinically manifest cardiovascular dysfunction [10–12]. Another important factor relating to the severity of AHR is the completeness of the spinal injury. Only 27 % of incomplete tetraplegics present with signs of AHR, in comparison with 91 % of tetraplegics with complete lesions [11]. Currently, still no consensus regarding anesthesia management of these patients. Many anesthetic techniques have been proposed and used with varying success, but none of them is uniformly successful [13]. It is well known that the development of intraoperative AHR and hypertension can be prevented either by a deep general anesthesia with potent volatile agents, which blunts autonomic reflexes, or regional anesthesia (spinal or epidural), which blocks afferent and autonomic efferent neural impulses [2, 13–15].

There was a severe lack of controlled trials in the management and prevention of AHR. A variety of options are available to prevent AHR (e.g., surgical, pharmacological), but only intersphincteric anal block with lidocaine when undergoing anorectal procedures had evidence using a control group (Level 1) [16]. The identification and elimination of specific triggers for AHR (e.g., distended bladder) are considered the first line of treatment based on physiological rationale and expert consensus. When non-pharmacological actions fail in an acute episode, pharmacological agents are required and nifedipine, nitrates, and captopril are the most commonly used and recommended agents. However, only nifedipine is supported by controlled trials (Level 2) [16].

In this case, we though in administering lidocaine by the fact that, first, due to drug properties and then, because there are reports that lidocaine anal block significantly limits the AHR response in susceptible patients undergoing anorectal procedures and cases of AHR treated successfully with epidural lidocaine [17]. To our knowledge, this is the first case report of severe AHR treated successfully with iv lidocaine. More studies to determine which of these agents, including intravenous lidocaine, or combinations of therapies are effective, are severely needed.

### Consent

Written informed consent was obtained from the patient for publication of this Case report and any accompanying images. A copy of the written consent is available for review by the Editor-in-Chief of this journal.

## Abbreviations

AHR: autonomic hyperreflexia
BP: blood pressure
HR: heart rate
PACU: post-anesthesia care unit
SCI: spinal cord injury
